# Antidiabetic Properties of Hydroalcoholic Leaf and Stem Extract of *Levisticum officinale*: An implication for α-amylase Inhibitory Activity of Extract Ingredients through Molecular Docking

**DOI:** 10.22037/ijpr.2020.15140.12901

**Published:** 2020

**Authors:** Nahid Ghaedi, Iran Pouraboli, Nayere Askari

**Affiliations:** *Department of Biology, Faculty of Sciences, Shahid Bahonar University of Kerman, Kerman, Iran*

**Keywords:** Diabetes mellitus, Levisticum officinale, Antioxidants, Glucose transporters, Phytochemicals, Molecular docking

## Abstract

*Levisticum officinale* (Apiaceae) is a favorite food spice. Iranian folk medicine claims that it has a prominent antidyslipidemic property but this is not documented scientifically so far. This study evaluated antidyslipidemic and the other antidiabetic aspects of the stem and leaf hydroalcoholic extract of it (LOE). Regarding oral glucose tolerance test results, LOE (500 mg/kg) administration 30 min before glucose loading significantly decreased the blood glucose level (13%) at 90 min in male rats. Additionally, LOE treatment (500 mg/kg, orally, once a day) for 14 days significantly reduced the serum glucose level (24.97%) and markedly improved the lipid profile and the insulin, creatinine, alanine aminotransferase and aspartate aminotransferase serum levels in diabetic rats. Moreover, LOE effectively amended the impaired antioxidant status and ameliorated lipid peroxidation in the plasma and pancreas and liver tissues of diabetics. Also, 14 days LOE treatment, significantly decreased the renal sodium-glucose cotransporter 2 and facilitated glucose transporter 2 (GLUT_2_) mRNA levels and GLUT_2_ gene expression in the enterocytes of jejunum tissue in comparison with diabetic untreated rats. HPLC method revealed the presence of chlorogenic acid, rosmarinic acid, caffeic acid, quercetin and luteolin and GC-MS analysis detected bioactive compounds like phthalides, thymol, phytol, hexanoic acid, carene and menthofuran. LOE showed α-amylase (αΑ) inhibitory activity and in silico studies predicted that among extract ingredients luteolin, quercetin, rosmarinic, caffeic, and hexanoic acids have the greatest αΑ inhibition potecy. Thus, current results justify antidyslipidemic value of *L. officinale* and shed light on more antidiabetic health benefits of it.

## Introduction

Diabetes mellitus (DM) is a serious prevalent metabolic disorder and a public health problem affecting a large proportion of population. It is well characterized by hyperglycemia, and dyslipidemia. Hypergly-cemia induces oxidative stress and leads to progression of DM-associated damages. Hence, blood glucose control is the main policy for DM management. Starch and the other carbohydrates of diet are hydrolyzed by pancreatic α- amylase (αA) and intestinal α- glucosidas enzymes and then resultant monosaccharides leave these cells toward the bloodstream through apical sodium- glucose transporter 1 (SGLT1) and basal facilitated glucose transporter 2 (GLUT2). Furthermore, lots of filtered glucose in the kidney’s glumeruli is reabsorbed via SGLT2 and GLUT2 transporters at luminal and basal epithelial membranes of proximal tubules, respectively. It seems that αA inhibition and glucose reuptake restriction are effective strategies for lowering blood glucose rise after meal. Despite availability of different classes of synthetic antidiabetic drugs, research in this field has continued because of undesired side effects of these known agents. Plants as a major natural source of antioxidants, have been a favorite source of drugs with fewer adverse side effects but a lot of health benefits. Medicinal plants have been used for DM remedy from a long times ago. *Levisticum officinale *Koch (Apiaceae), or lovage, is locally called *karafs-e koohi* in Iran, grows in a humid valley beside the Ordigan waterfall at 3200-3500 m in elevation on Hezar mountain in Kerman province. This plant is a cultivated or a semi-native plant in Europe and North America (1). *L. officinale *is a perennial and aromatic plant with a celery-like flavour and smell. Different parts of *L. officinale* have been used for many medicinal purposes. Its root has been used as carminative, spasmolytic (2), and diuretic (3). The antimicrobial and antioxidant activity of the hexane extracts from the roots and seeds of *L. officinale* have been determined (4) and it was revealed that a methanol extract of *L. officinale *root showed acetyl cholinesterase and pancreatic lipase inhibitory activity (5,6). Also, the methanolic and aqueous extract of *L. officinale* root showed a strong inhibitory effect on α-glucosidase (7). Furthermore, the ethanol extract from the fruit of *L. officinale* induced apoptosis in leukemia cell lines (8) and ethanolic extract of aerial parts exerted antiproliferative effect against breast cancer cell lines (9). In the other hand, it is reported that aerial parts essencial oil of *L. officinale *has antioxidant (10) antibacterial (11) and antifungal (12) properties. Moreover, ethanolic extract of its leaves, stem and root had radical scavenging activity (13). The stem and leaves of this plant are used as spice for milk products in Iran and despite local people acclaim on strong antidyslipidemic property of it, this has not been scientifically documented so far. Since, no study has been designed thus far to investigate the effect of hydroalcoholic extract of the stems and leaves of *L. officinale* (LOE) on various aspects of diabetic complications including dyslipidemia, the objective of this study was conducted to determine this and the related probable mechanisms and provide more valuable informations about bioactive constituents of LOE. Also, recent study evaluated and predicted αA enzyme inhibitory activity of extract ingredients by in silico analysis. 

## Custom

*Preparation of extract*

Fresh stems and leaves of *L. officinale* were collected during May and June from Hezar mountain in Kerman province in southeastern Iran. The plant was authenticated by a plant taxonomist, Dr. S.M. Mirtadzadini. A specimen was kept in MIR herbarium (voucher no. 2046) of Biology Department at Shahid Bahonar University of Kerman. The plant parts were dried at room temperature, ground to powder and extracted with 80% methanol by maceration. For this, powdered plant materials were allowed to soak in a closed container with solvent at room temperature for at least 3 days with occasional shaking. Then, the liquid part strained off and separated by filtration and was dried in 40 °C oven and stored at 4 °C for use (14). The final extraction yield was about 22%. 

*Animals and Experimental design*

Male Wistar rats weighing 200–250 g were housed in the animal room of the Department of Biology in cages and maintained under environmentally controlled conditions (23 ± 2 °C; 12/12 h light/dark cycle; 55% humidity). They were provided with free food and tap water access. The study procedure was approved by the Institutional Animal Ethics Committee of Shahid Bahonar University of Kerman (approval number: 1394). Diabetes was induced in 30 rats by intraperitoneal injection of 65 mg/kg, body wt. of streptozotocin (STZ) dissolved in citrate buffer (0.1 M; pH = 4.5). Hyperglycemia developed about five day STZ post-injection. The rats were fasted for 12 h and blood obtained from the tail vein for glucose level estimation by glucometer (Accu-check; Roche Diagnostics; Germany). The animals with fasting blood glucose levels above 300 mg/dl were considered to be diabetic. The diabetic rats were randomly divided into five groups (n = 6) and received distilled water, different doses of LOE (100, 300 and 500 mg/kg body wt.) and glibenclamide (20 mg/kg body wt.) by gavage for 14 days. One group of normal rats also treated with distilled water for 14 days. At the end, the fasted rats were anesthetized by inhalation of CO_2_ and killed by decapitation (15, 16). The blood samples were immediately collected and centrifuged for 10 min at 3000 *g* to obtain serum and plasma samples, which were then stored at -20 °C for analysis of biochemical parameters. Pancreas, liver, kidney, and jejunum samples of each group were excised and frozen at -70 °C. Serum glucose, triglycerides, total cholesterol, HDL-C, creatinine, aspartate aminotransferase (AST), and alanine aminotransferase (ALT) levels were measured using standard kits (Pars Azmoon; Iran) by spectrophotometer (CS-T240; ADVANCE; China) and LDL levels were calculated according to this equation: LDL = Total cholesterol - [HDL + (Triglycerides/5)]. Serum insulin levels were determined using rat enzyme immunoassay kits (Mercodia; Uppsala; Sweden).

*Oral glucose tolerance test (OGTT)*

Another 30 fasted normal male rats were divided into five groups (n = 6) and received distilled water, LOE (100, 300, and 500 mg/kg body wt.) and glibenclamide (20 mg/kg body wt.) individually by gavage 30 min before glucose loading (5 g/kg body wt.). The blood samples were collected from the tail vein before treatment and glucose loading as well as at 30, 60, 90, 120, and 240 min post-treatment and glucose serum levels determined (17).

*Antioxidant enzyme activity and lipid peroxidation measurement*

The pancreas and liver tissues were homogenized and centrifuged and the supernatants were used for antioxidant assessment. Catalase (CAT) activity was measured as described by Aebi (18). Superoxide dismutase (SOD) activity was characterized by the method recommended by Giannopolitis and Ries (19). The tissue protein levels were determined as described by Bradford (20). The malondialdehyde (MDA) levels in these tissues were measured as recommended by Ohkawa et al. (21) and modified by Jamall and Smith (22). The plasma MDA level was determined as described by Kurtel et al. (23).

*Determination of gene expression level by relative quantitative real-time RT-PCR*

Total RNA extraction from the jejunum and cortical portions of kidney tissue samples from different groups was done using a Total RNA Purification Kit (GeneAll; Korea). The purity and concentration of extracted RNA was determined by spectrophotometer (Nanodrop 2000c; Thermo Fisher Scientific; USA). The integrity of the RNA samples containing GelRed was detected on 1.5% agarose gel. A specific amount of total RNA samples were used for cDNA synthesis through RevertAid First Strand cDNA Synthesis kit (Thermo Fisher Scientific; UK) according to the manufacturer instructions. The primer sequences for desired genes were according to Honari et al. design (16)^.^and included SGLT1 (Forward: 5ʼ- CCTGTACCGTCTGTGC-3ʼ; Reverse: 5ʼ- ACAGAACAGGTCATATGCCTT-3ʼ), SGLT2 (Forward: 5ʼ- CCAATAGAGGCACAGTTGGTGG-3ʼ; Reverse: 5ʼ- GTAAATGTTCCACAACGG-3ʼ) and GLUT2 (Forward: 5ʼ- TACATTGGCGAGATTGCT-3ʼ; Reverse: 5ʼ- TGATTGCCCAGAATGAAG-3ʼ) and β-actin as an internal control (Forward: 5ʼ- GCTGAGAGGGAAATCGTGC-3ʼ; Reverse: 5ʼ-TCAACGTCACACTTCATGATGG-3ʼ). Also, the products sizes of these were 111, 186, 122 and 253 bps, respectively. To determine the sodium-glucose cotransporter 1 and 2 (SGLT_1_, SGLT_2_) and facilitated glucose transporter 2 (GLUT_2_) mRNA levels, relative quantitative real-time PCR was done with a multicolor real-time PCR detection system (iQ5; Bio-Rad; USA). The relative amount of mRNA was calculated by the Livak method (24) as: fold change or relative Expression = 2^-ΔΔCt^.

*Evaluation of pancreatic α-amylase inhibition potency of LOE*

To evaluate the inhibitory effect of extract on pancreatic α-amylase activity, Porcine pancreatic α-amylase enzyme solution (ppA; 0.5 U/mL) and various concentrations of LOE (0.1, 0.5, 1, and 10 mg/mL in DMSO) were mixed and incubated at 25 °C for 30 min. Next, a starch solution (0.5%) was added and incubated at 37 °C for 15 min. At the end, color reagent solution included DNSA (96 mM) was added and placed into a water bath at 85 °C for 15 min. The reaction mixture absorbance value was read at 540 nm against associated blank after dilution with distilled water (25). The control tube (without extract) was considered to be 100% enzyme activity. Acarbose (Exir; Iran) (0.5, 1, 2, 5, 10 mg/mL), the known ppA inhibitor, was used as the positive control. The inhibition percentage of α-amylase determined according to the following formula:

**I **_α__- __amylase_ (%) = 100 × [ΔA_Control _– ΔA_Sample_] / ΔA_Control_

*Phytochemistry analysis of extract*

Preliminary phytochemical screening of LOE was carried out by the usual chemical tests for characterization of different plant metabolites (26). The method described by Singleton et al. (27) was used to measure the total phenolic compound content in the LOE with some modifications. Data were reported as milligrams of Gallic acid equivalent per gram of dry weight of extract (GAE/g). The method designed by Kumazawa et al. was used to optimize and as a guideline for this assay. In this method, the quantification of flavonoids is based on its complex product with aluminum chloride solution (10% w/v) and the spectrophotometric determination of the formed complex. Data were recalculated as milligrams of catechin equivalent per gram of dry weight of LOE (28). 

HPLC system was applied to determine the amount of some of the flavonoids and phenolic acids in the LOE. For this, the chromatographic system conditions were set at room temperature (25 °C), quercetin (Q) ≥ 95.0%, luteolin > 98%, chlorogenic acid (CGA) ≥ 95.0%, rosmarinic acid (RA) > 98% and caffeic acid (CA) > 98% were used as standards and UV detector (K2501) adjusted at 330 nm. Also, mobile phases including A: 15% methanol (adjust to pH 2 with phosphoric acid) and B: methanol were used for CGA and CA, mobile phases including A: 0.3 g/L solution of phosphoric acid (adjust to pH 2) and B: methanol for Q and luteolin and mobile phases including A: phosphoric acid/acetonitrile/water (1:19:80 v/v/v) and B: phosphoric acid/methanol/acetonitrile (1:40:59 v/v/v) for RA were applied. The flow rate was 1.2 mL/min for RA and 1 mL/min for the others and reversed-phase C18 column (5.0 μm, 250 mm in length, 4.6 mm inner diameter) was used. Crude extract was dissolved in methanol 80%, passed through 0.45 μm filter and injected to HPLC system. The injection volume was 20 µL. Chromgate software used for data acquisition.

Also, above filtered LOE was subjected to gas chromatography and mass spectroscopy (GC-MS) analysis for phytochemical screening of bioactive volatile and semi-volatile compounds. This was carried out using Agilent 6890 series equipped with 5973 Mass Selective Detector, HP-1 capillary column of dimensions 30.0 m × 250 μm × 0.5 μm and helium (99/9%) as carrier gas with a flow rate of 1 mL/min. The oven temperature was set initially at 50 ºC for 2 min, followed by an increase of 8 ºC /min to 250 ºC and was kept isothermally for 30 min. The split sampling technique was used to inject 1 µl of sample in the ratio of 1:5. The quadrapole MS was operated at 70 eV and MS spectra range was 40-500 m/z. LOE constituents were identified and interpreted by comparison of their mass spectral data including gas chromatogram retention times with those from NIST and Wiley libraries. 

*Molecular docking (In silico studies)*

Docking studies through Virtual screening technique were designed with some of known extract constituents (as ligands) with human pancreatic α- amylase enzyme (as receptor) in order to determine or confirm possible role of known extract ingredients in α- amylase enzyme inhibitory potency of extract. Virtual screening is a computational alternative method in the drug discovery field and has had a great success in this pharmaceutical field (29, 30). For this, human pancreatic α- amylase enzyme crystal structure (PDB ID: 5U3A) was retrieved from the RCSB protein data bank (PDB) and this 3D structure was prepared and optimized by removing water and other excess molecules or atoms before docking and the outputs were saved for docking process. Moreover, the 3D structure of the known desired extract ingredients, including chlorogenic acid, rosmarinic acid, caffeic acid, quercetin, luteolin, phthalides, thymol, phytol, hexanoic acid, carene, and menthofuran were downloaded from Zinc 15 database and designed in the SDF format. Conformational analysis and geometry optimization and preparation of both receptor (α-amylase enzyme) and ligands (above known extract compounds) were done. Schrodinger 2018-4 software package was used for all steps of this docking study. With respect to docking outputs, a more negative docking score means a more and stronger interaction possibility between associated ligand and α- amylase enzyme. Docking output results were visualized using LigPlot^+^. 

*Evaluation of LOE toxicity*

Healthy Wistar rats were fasted for 12 h, then treated with an oral dose of LOE (5000 mg/kg body wt.). The animals were followed up for behavioral and neurological signs of toxicity (fatigue, paw licking, watery stool, writhing, and loss of appetite) and mortality for up to 72 h. Also, the blood metabolic parameters were monitored at the end of this period.

*Statistical analysis*

The results were reported as mean ± SEM. One way analysis of variance (ANOVA) and Tukey post test were applied for statistical analysis. The differences were accepted significant at *p* < 0.05.

## Results and Discussion

In this study, an acute toxicity test with a pharmacological procedure was used for the determination of LD_50_ and the selection of safe dosage range of LOE used in this study. Results showed no mortality or sign of toxicity at 5000 mg/kg was caused by acute administration of LOE after 72 h. Hence, the LD_50_ of LOE is above this value and the recent applied doses of LOE (100, 300 and 500 mg/kg) can be considered safe. 

**Figure 1:**
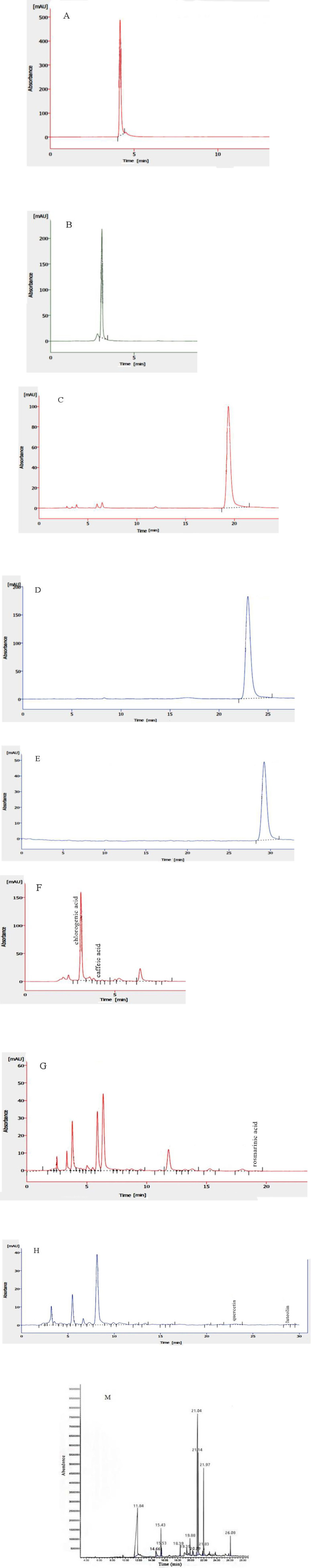
HPLC chromatograms obtained from standards including A: caffeic acid, B: chlorogenic acid, C: rosmarinic acid, D: quercetin, E: luteolin and LOE samples (F, G, H)**. **GC-MS spectral chromatogram of LOE (M).

It is well established that plant extracts possess different bioactive substances which lead to their different properties, so, here, we performed phytochemical analysis of LOE in order to determine what the extract contains. Qualitative phytochemical screening of LOE showed the presence of flavonoids, phenols, tannins, glycosides, steroids, and phytosterols. Also, according to quantitative approaches, the phenol and flavonoid content in the LOE were 69.78 ± 9.3 and 36.17 ± 4.19 mg/g of extract dry weight, respectively, based on the Gallic acid and catechin concentration/absorption curves. In addition, HPLC analysis of LOE revealed the presence of 188.73 ± 2 mg/g CGA, 0.26 ± 0.01 mg/g rosmarinic acid, 0.028 ± 0.003 mg/g caffeic acid as phenolic acids and 0.36 ± 0.05 mg/g quercetin and 0.21 ± 0.005 mg/g luteoin as flavonoids (Figure 1 A-H). Moreover, in the GC-MS analysis, the mass spectra of identified chemical compounds from LOE were matched with those found in the NIST/Wiley libraries are listed in Table 1 and the associated chromatogram is depicted in Figure 1, M. However, in this, the peaks at 15.53 min and 18.39 min are related to the solvents. Results of this analysis of LOE revealed the presence of phytochemical compounds like phthalides, thymol, phytol, hexanoic acid, carene and menthofuran which include important medicinal properties. 

Regarding the literature, phytochemical analysis of essential oils of different parts of *L. officinale* determined the presence of monoterpenic hydrocarbons as β-phellandrene and α-Terpinyl acetate (31), Curzerene γ-Cadinene, Sabinene (10), 6-butyl-cyclohepta-1,4-diene and 7-formyl-4-methyl-cumarine (12), pentyl cyclohexa-1,3-diene, Z-ligustilide, neocnidilide, Z-β-ocimene, p-menth-1-en-8-ol acetate and pentyl cyclohexa-1,3-diene (11, 31). Besides, Tomsone et al. reported that there are the highest content of phenolic and flavonoid compounds in root, seeds and especially in lovage leaves ethanolic extract and confirm partly some of our above results (13). Thereby, even though there are informations about the composition of *L. officinale* essential oil, this was the first study to describe and partially quantify phytochemical components of hydroalcoholic extract of stems and leaves of lovage (LOE) with above valid methods.

In DM, disturbances in body metabolic regulatory mechanisms caused by insulin deficiency or insulin resistance led to dyslipidemia due to recruitment of free fatty acids from peripheral fat stores. This increases the production of triglyceride-rich lipoproteins in the liver were accompanied by a decrease in HDL-C (32). An increase in serum ALT and AST levels is mainly caused by leakage of these enzymes from liver cells into the blood stream and indicates the loss of hepatocyte integrity in DM (33). In the present study, anti-diabetic properties of different doses of LOE (100, 300 and 500 mg/kg) were determined. Results showed that LOE (500 mg/kg) administration, for 14 days significantly decreased serum glucose level (24.97%) and increased insulin serum level compared to the diabetic rats. Also, LOE treatment (300 and 500 mg/kg) significantly decreased the level of serum cholesterol, triglycerides, LDL, creatinine, ALT, AST and increased the HDL levels. Moreover, LOE (100 mg/kg) caused a decrease in the total cholesterol, LDL and creatinine serum levels compared with the control diabetic rats (Table 2).

Moreover, in the current experiment, administration of LOE (100, 300 and 500 mg/kg) and glibenclamide as a reference drug (20 mg/kg) on the glucose tolerance in normal rats are shown in Table 3. At 90 min after glucose loading or 120 min after LOE administration (500 mg/kg), the blood glucose level had decreased significantly by 13% when compared with the control rats. 

These results can be attributed to above extract ingredients because, it has been shown that CGA exerts hypoglycemic and hypolipidemic effects (34) and it is able to modulate glucose uptake and stimulate insulin secretion from rat Langerhans islets (35). Moreover, rosmarinic acid (RA) has a significant hypoglycemic effect and insulin secreting activity in diabetic rats (36). Also, quercetin is able to regenerate the pancreatic islets and induce insulin release. Additionally a significant decrease in glucose, cholesterol, triglycerides, and glucose absorption levels have been observed in quercetin-treated diabetic rats (37). Furthermore, thymol and phytol have antihyperglycemic and antihyperlipidemic properties (38, 39) and hexanoic or caproic acid improves insulin stimulated glucose uptake (40).

It is known that glucose uptake increases in DM. SGLT1 in the apical membrane and GLUT2 in the basal membrane of enterocytes in the small intestine are mainly involved in the glucose absorption after carbohydrate digestion. Moreover, glucose transporters in the proximal tubule of kidney (SGLT2 and GLUT2) are critical to glucose reabsorption. In uncontrolled diabetes, adaptive changes caused by hyperglycemia increase the flux of transepithelial glucose through renal (41) and intestinal glucose transporters (42).

**Figure 2:**
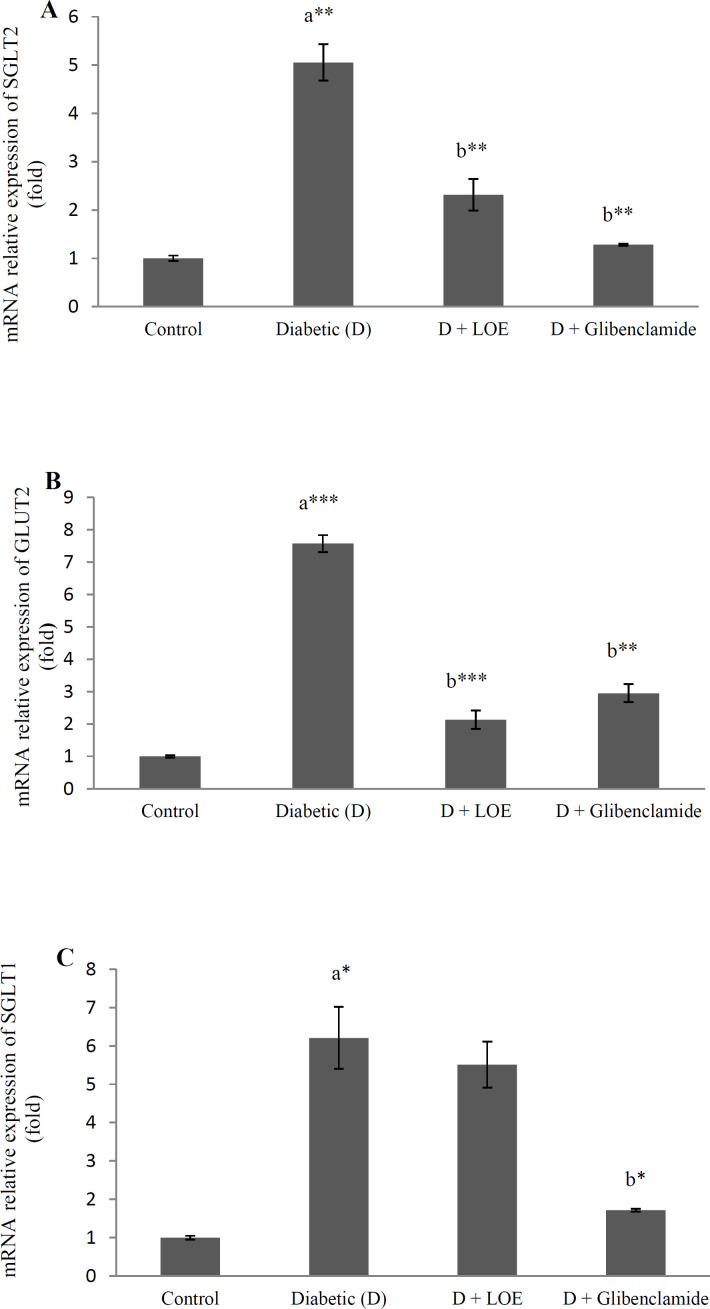
The effect of LOE on SGLT1 (A), and GLUT2 (B) mRNA relative expression in the kidney tissue and SGLT2 (C) and GLUT2 (D) mRNA relative expression in the jejunum tissue of diabetic rats. Data were presented as mean ± SEM (𝑛 = 6).

^a^ compared to control group.

^b^ compared to diabetic group.

Here the effect of LOE (500 mg/kg) on glucose transporter gene expression in the jejunum and kidney tissue of diabetic rats were investigated in order to find out the antihyperglycemic mechanism of extract more, Results showed that renal SGLT2 and GLUT2 (Figure 2 A, B) as well as jejunum SGLT1 and GLUT2 gene expression levels (*p* < 0.05) (Figure 2 C, D) increased significantly in the diabetic rats compared to the control groups and LOE (500 mg/kg) administration significantly decreased the renal SGLT2 (*p* < 0.01), GLUT2 (*p *< 0.001), and jejunum GLUT2 mRNA levels (*p* < 0.001) (Figure 2).

 It has been reported that CGA maintains glucose homeostasis by modulating the expression of SGLT-1 and GLUT2 in the intestinal segments of rats fed with a high-fat diet (43).

Moreover, a decrease in glucose absorption by caffeic acid (CA) through the inhibition of intestinal SGLT1 and GLUT2 has been reported (44). Moreover, quercetin acts as an antidiabetic agent through glucose absorption reduction in quercetin-treated diabetic rats (37). It has been reported that inhibition of glucose absorption by quercetin can occur through competitive inhibition of SGLTs or by noncompetitive inhibition of intestinal transporter GLUT2 (45).

One of the other therapeutic strategies to decrease the blood glucose levels in DM is inhibition of the oligo and disaccharides breakdown to absorbable monosacarides. This could be done by inhibition of the carbohydrate-hydrolysing enzymes like α-amylase (Αα). Pancreatic α-amylase enzyme catalyzes carbohydrate breakdown to oligosaccharides and disaccharides in the intestine. It is previously justified that α-amylase inhibitors can improve hyperglycemia in diabetic conditions (46). It is well documented that plant polyphenoles are inhibitors of this enzyme which can reduce glucose absorption and blunt the plasma glucose rise (47, 48).

Recent findings showed that LOE has 14% ± 2% α-amylase inhibitory activity at a concentration of 10 mg/ml compared to the inhibitory effect of acarbose (94% ± 3%) as a reference drug. This result can be attributed to the presence of phytochemical compounds especially phenolic compounds in the extract with well known α-amylase inhibitory effect as** p**revious findings reports that RA (49), luteolin and quercetin (47) have pancreatic α-amylase inhibitory effect. Also, Komaki et al. reported that luteolin glucosides have anti- human pancreatic α-amylase property (50). Finally, our recent *in silico* study investigated the probable α- amylase enzyme inhibitory activity of 11 known phytochemical compounds of LOE to predict possible antihyperglycemic potency of them. Regarding the resultant docking scores (Table 4), among LOE docked compounds, luteolin, quercetin, rosmarinic, caffeic, and hexanoic acids had the greatest interaction probability with α- amylase enzyme then, thymol, carene, phthalides, and phytol showed enzyme interactions more weakly, respectively. Also, chlorogenic acid and menthofuran as the other LOE ingredients show no interaction through a molecular docking simulation (Table 4). 

**Figure 3:**
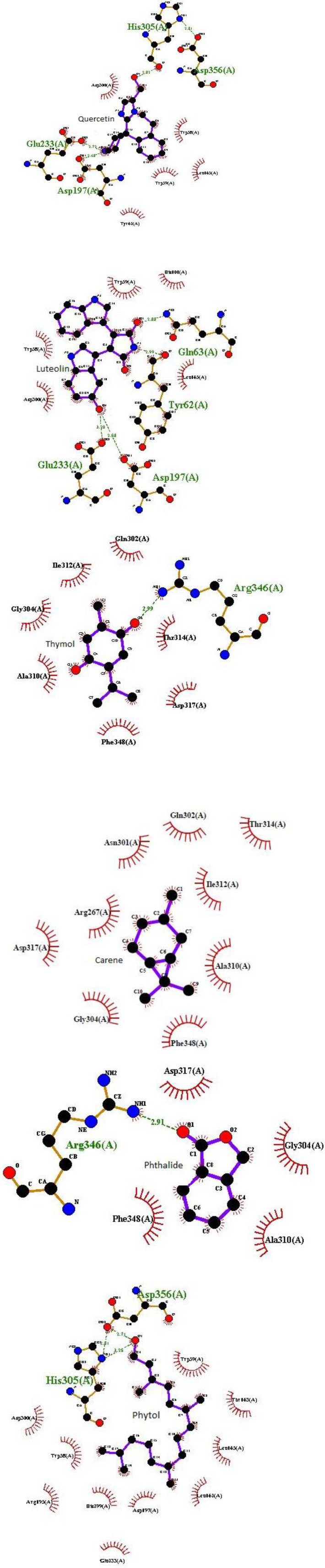
Schematic illustration of specific interactions between LOE constituents and human pancreatic α- amylase (PDB ID: 5U3A). Graphics obtained using Ligplot^+^.^.^

As shown in this table, molecular docking output revealed that quercetin, rosmarinic, and hexanoic acids have a similar interaction core with αA enzyme. Also, luteolin and caffeic acid are the same in interaction loci with this enzyme. The α- amylase enzyme is a single-chain protein which has three structural domains and amino acids residues 1-99 and 69-404 in domain A houses its active site. The active site of it includes catalytic residues: Asp197, Glu233, and Asp300 (51). Intermolecular interactions obtained from docking algorithms structural analysis were presented in Figure 3. 

Regarding this, Asp197 and Glu233 at catalytic active site of αA are critical amino acid residues which get involved in at least five of the ligands (luteolin, quercetin, rosmarinic, caffeic and hexanoic acids) which have the strongest interaction with αA. Then, Arg346 in thymol and phthalides and His305 in quercetin and phytol also, can consider as important interacting residues. 

In line with our molecular docking results, Rasouli *et al.* demonstrated possible α- amylase enzyme inhibitory activity of caffeic acid, luteolin and quercetin by virtual screening method and described that the αA inhibitory activity of luteolin was stronger than luteolin glucoside (52). Thus, according to these in silico results, we can confirm antihyperglycemic property of luteolin, quercetin, rosmarinic acid, and suggest caffeic and hexanoic acids, thymol, carene, phthalides, and phytol as potential eligible lead components to inhibit α- amylase enzyme and predict an effective antihyperglycemic potential for them. However, complementary experimental studies needed to verify this. In addition, molecular dynamic results from recent work can be helpful for future studies about drug design against DM.

There are a lot of reports include interactions between diabetes and oxidative stress. A decrease in SOD and CAT activity in the liver and pancreas of diabetic rats has been reported (53). SOD and CAT enzymes are major components of the antioxidant defense system of the body. Decreased activity in antioxidant enzymes increases accumulation of free radicals, which in turn leads to lipid peroxidation. This is determined by the elevation of MDA content. MDA can damage body organs such as the liver and pancreas (54). 

In this study, the enzymatic SOD and CAT activities in the pancreas and liver tissues decreased significantly (p < 0.05) in the diabetic rats and LOE administration (500 mg/kg) increased these tissue enzymes activities in the diabetic treated rats in comparison with the untreated ones (p < 0.01; Table 5). Diabetic animals exhibited a significant increase in the plasma, pancreas and liver tissues MDA levels (p < 0.001) and LOE (500 mg/kg) significantly decreased the MDA levels over that of the controls (p < 0.001; Table 5).

As mentioned above, there are many polyphenolic compounds with antioxidant activity in the extract which mediate these properties. Kono *et al.* (55) had shown that chlorogenic acid and caffeic acids exert antioxidant property. Also, luteolin as another flavonoid in LOE possess antioxidant and anti-inflammatory properties (56) and menthofuran is an antioxidant with radical scavenging activity (57). Rosmarinic acid (RA) is a phenolic acid known as a potent antioxidant (58).

Thus, these substances are closely associated to antioxidant effect of LOE.

*p < 0.05; **p < 0.01; ***p < 0.001

**Table 1:** Identified LOE chemical constituents from a GC-MS analysis

NO.	Retention Time (min)	Compounds	Peak area (%)
1	11.843	Hexanoic acid	29.81
2	14.667	Thymol	1.69
3	15.434	Carene	2.79
4	19.395	Isobutylidenphthalide	1.27
5	19.882	Cyclopropane, 1-ethenyl-2-hexeny	2.25
6	20.289	Butylidene Phthalide	0.37
7	21.045	Isobutylidenphthalide	35.89
8	21.135	n Butyl Phthalide	11.92
9	21.833	Menthofuran	0.56
10	21.976	n Butyl Phthalide	11.5
11	26.080	Phytol	1.9

**Table 2:** Effect of 14 days administration of different doses of LOE on serum levels of biochemical factors.

Groups	Glucose (mg/dl)	Insulin (µmol/ml)	Triglycerides (mg/dl)	Total cholesterol (mg/dl)	HDL (mg/dl)	LDL (mg/dl)	Creatinine (mg/dl)	AST (IU/L)	ALT (IU/L)
Control	85.6 ± 2.4	4.4 ± 0.01	67.5 ± 3	77 ± 3.2	41.1 ± 2.2	22.5 ±2	0.9 ± 0.0	137 ± 6	74 ± 4.7
Diabetic (D)	467 ± 6^a***^	1.08.0 ± 0.02^a***^	93.0 ± 6^a**^	108 ± 3.7^a***^	24.1 ± 1.5^a***^	65.4 ± 3^a***^	1.3 ± 0.01^a***^	238 ± 15^a***^	188 ± 6^a***^
D + Extract (100 mg/kg)	453 ± 6	2.0 ± 0.02	80.7 ± 1	82 ± 3.8^b***^	29.2 ± 0.8	36.86 ± 2.4^b**^	0.9 ± 0.0^b**^	235 ± 3	190 ± 3.8
D + Extract (300 mg/kg)	430 ± 6.7	2.0 ± 0.0	61.7 ± 6^b**^	44.5 ±3.5^b***^	32.5 ± 2.9^b*^	19.66 ± 3.2^b***^	0.5 ± 0.01^b***^	173 ± 4^b**^	147 ± 16^b*^
D + Extract (500 mg/kg)	350 ± 16^b***^	2.1 ± 0.01^b*^	72.3 ± 1^b*^	48.6 ± 3.3^b***^	38 ± 3.4^b**^	16.14 ± 1.7^b***^	0.5 ± 0.02^b***^	144 ± 10^b***^	57 ± 6.8^b***^
D + Gliben. (20 mg/kg)	179 ± 13^b***^	2.3 ± 0.01^b***^	62.0 ± 2^b**^	52.2 ± 1.7^b***^	38 ± 1.2^b**^	11.8 ± 2.2^b***^	0.6 ± 0.03^b***^	127 ± 3^b***^	66 ± 1.3^b***^

Values are given as mean ± S.E.M, n=6. ^a^ compared to control group.

^b^ compared to diabetic group. *p < 0.05; **p < 0.01; ***p < 0.001.

**Table 3:** Effect of different concentrations of LOE on glucose tolerance in normal rats

Groups		Glucose (mg/dl)				
	0 min	30 min	60 min	90 min	120 min	240 min
Control	92.6 ± 4.0	133.0 ± 1.1	136 ± 2.0	127 ± 1.5	113 ± 3.2	92.3 ± 5.4
Extract (100 mg/kg)	92.0 ± 4.3	143.3 ± 3.8	141 ± 2.7	140 ± 2.8	137 ± 2.6	100±4.1**
Extract (300 mg/kg)	89.3 ± 4.6	135.3 ± 10.8	136 ± 2.0	130 ± 1.7	120 ± 9.3	105 ± 8.0
Extract (500 mg/kg)	80.3 ± 3.7	125.0 ± 13.5	125 ± 7.6	110 ± 5.7*	107 ± 4.9	95 ± 2.6
Glibenclamide (20 mg/kg)	74.0 ± 3.0*	105 ± 2.5**	101 ± 0.8**	97 ± 2.0***	95.6 ± 2.9	84 ± 3.3

Different doses of LOE administered and 5 g/kg b.w. glucose loaded 30 min after it. Blood glucose levels determind at 0, 30, 60, 90, 120, and 240 min of OGTT. Each value represents mean ± SEM, n = 6 rats per group.

**p < 0.01, ***p < 0.001 (compared with control group).

**Table 4:** The docking scores, interaction loci and binding types of different known extract (LOE) compounds (ligands) with human pancreatic α- amylase (PDB ID: 5U3A) as receptor. All distance values are in angstrom (A°).

	Extract ingredients										
	Rosmarinic acid	Quercetin	Hexanoic acid	Luteolin	Caffeic acid	Thymol	Carene	Phthalide	Phytol	Menthofuran	Chlorogenic acid
**Docking score**	-7.85	-7.85	-7.65	-7.6	-7.4	-3.4	-3.2	-2.9	-1.84	-	-
**Interaction** (Type, Distance, Site)	H-bond distance:1.92 His305 H-bond distance:1.78 Glu233 H-bond distance:1.68 Asp197 Salt bridje distance:2.72 Glu233 Salt bridje distance:3.93 Asp197			H-bond distance:1.64 Asp197 H-bond distance:2.42 His299 H-bond distance:2.03 Gln63 H-bond distance:2.02 Tyr62 Pi-Pi stacking distance:4.19 Trp59		H-bond distance:2.07 Arg346	Mild	H-bond distance:1.9 Arg346	H-bond distance:1.85 Asp356	-	-

**Table 5:** Effects of LOE on antioxidant parameters in plasma, pancreas and liver tissues of diabetic rats

Groups	SOD activity (U/mg protein)		CAT activity (U/g protein)		MDA (nmol/g tissue)		Plasma MDA (nmol/mL)
	Pancreas	Liver	Pancreas	Liver	Pancreas	Liver	
Control	16.7±0.4	11.2±0.2	48.4±2	209.9±1.7	0.4±0.0	3.2±0.2	0.9±0.0
Diabetic (D)	11.2±0.6^a***^	8.0±0.8^a*^	28.8±1.3^a***^	103.5±15.4^a***^	1.7±0.0^a***^	12.8±0.6^a***^	5.6±0.1^a***^
D + Extract (500 mg/kg)	16.6±0.5^b***^	14.8±0.4^b***^	43.0±1.9^b**^	163.6±1.7^b**^	0.6±0.0^b***^	3.6±0.1^b***^	1.7±0.1^b***^
D + Glibenclamide (20 mg/kg)	16.9±0.3^b***^	16.2±0.7^b***^	44.6±1.4^b**^	167.4±1^b**^	0.5±0.0^b***^	3.4±0.1^b***^	1.6±0.1^b***^

Values are given as mean ± S.E.M, n = 6.

^a^ compared to control group.

^b^ compared to diabetic group.

*p < 0.05; **p < 0.01; ***p < 0.001

## Conclusion

In summary, the results of the present study show that LOE has significant antidyslipidemic and antioxidant properties and in silico studies predicted α- amylase inhibiton potency for some of its phytochemicals which may be responsible for antihyperglycemic effect of it. Also, in the light of these findings, we can justify *Levisticum officinale* (lovage) usage as an effective alternative medicine for diabetes complications such as dyslipidemia. 
